# A New Diagnosis of Ulcerative Colitis on a Background of Diversion Colitis Following Hartmann's Procedure for Perforated Diverticula

**DOI:** 10.7759/cureus.73837

**Published:** 2024-11-17

**Authors:** Aravind Sunderavel Kumaravel Kanagavelu, Aria Khani, Roxanne Boloorsazmashadi, Rimsha Khan, Louise Langmead, Shameer Mehta

**Affiliations:** 1 Gastroenterology, Barts Health NHS Trust, London, GBR

**Keywords:** diversion colitis, hartmann's procedure, inflammatory bowel disease, ulcerative colitis, vedolizumab

## Abstract

Ulcerative colitis (UC) is a chronic immune-mediated intestinal condition. This case report describes an 82-year-old woman who was newly diagnosed with UC. Two years prior, she had multiple admissions for abdominal pain and rectal bleeding, initially diagnosed as diverticulitis. Following Hartmann's procedure for a sealed perforation, she developed diversion colitis. Subsequent symptoms included abdominal pain, vomiting, and high stoma output, with CT imaging revealing upstream colitis and para-stomal cellulitis. Biopsies confirmed severe inflammation and ulceration consistent with UC. The patient was treated with intravenous hydrocortisone, but her symptoms rebounded upon steroid tapering. Vedolizumab was considered due to her venous thromboembolism (VTE) risk. Post-treatment, her stoma output and skin irritation improved. This case supports the theory that diversion colitis may predispose individuals to developing UC.

## Introduction

Ulcerative colitis (UC) is a chronic immune-mediated intestinal condition, although the exact etiology and pathophysiology are not fully understood [1]. The incidence of UC is highest between the ages of 20 and 40, and it is strongly associated with a positive family history and non-smoking [1]. Previous case reports describe diversion colitis as a possible risk factor for UC [2-4]. Here, we present a rare case of UC developing on a background of diversion colitis following Hartmann's procedure for perforated diverticula, suggesting that diversion colitis may be a risk factor for UC. Medical treatment appears effective, while surgery can also be considered.

## Case presentation

This case report describes an 82-year-old woman with a new diagnosis of UC. The patient has a background of polymyalgia rheumatica, hypertension, recurrent pulmonary embolism (PE) on warfarin, sickle cell trait, chronic kidney disease, and primary hyperparathyroidism. She is a non-smoker and consumes minimal alcohol socially.

**Figure 1:**
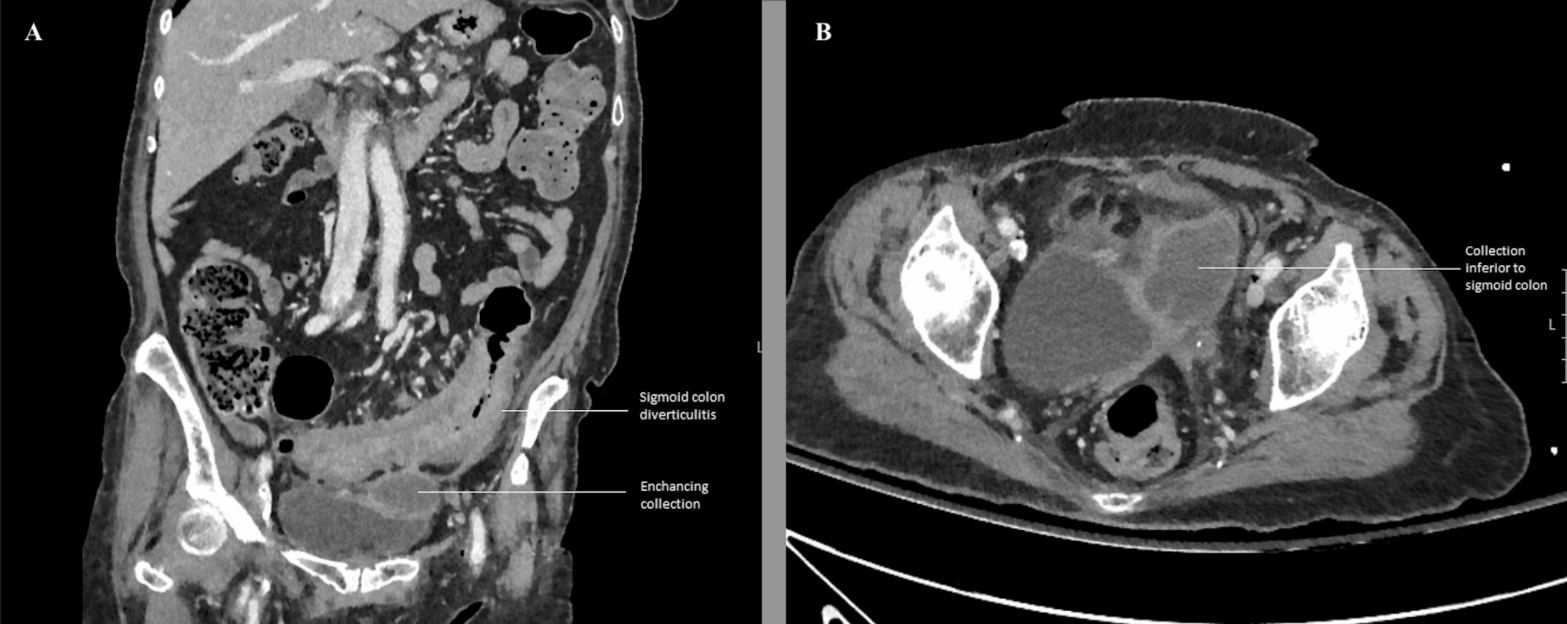
Computed tomography (CT) of the abdomen and pelvis showing signs of diverticulitis with collection (A) Coronal CT section showing sigmoid diverticulitis with enhancing collection; (B) axial CT section showing collection inferior to the sigmoid colon

Two years prior, the patient had four admissions due to episodic abdominal pain and rectal bleeding. At that time, computed tomography (CT) of the abdomen and pelvis, along with CT colonoscopy, revealed signs of diverticulitis with collection, which was managed conservatively with oral antibiotics and supportive measures (Figure 1). There were no historical fecal calprotectin values, nor had she previously undergone a colonoscopy. Subsequent admissions were due to stricturing diverticular disease and ultimately leading to a sealed sigmoid perforation. Consequently, she underwent Hartmann's procedure with an end colostomy without complications.

**Figure 2:**
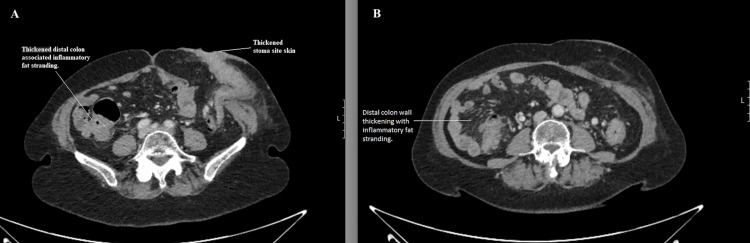
Computed tomography (CT) of the abdomen showing colitis of the distal colon with stomal cellulitis (A) Axial CT section showing colitis with inflammatory fat stranding and thickening at the stoma site; (B) axial CT section showing inflammatory fat stranding

**Figure 3:**
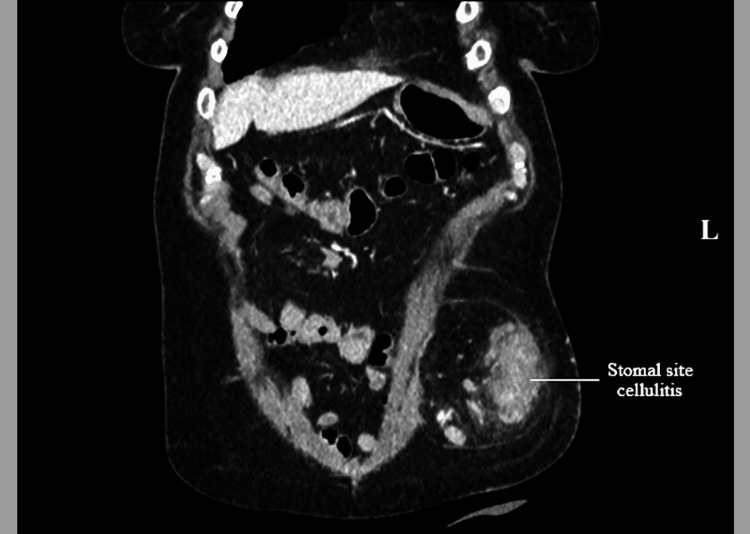
Computed tomography (CT) of the abdomen showing stomal site cellulitis

**Figure 4:**
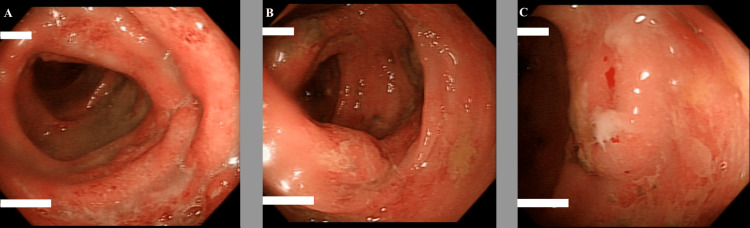
Image taken at sigmoidoscopy, performed through the stoma, showing an inflamed and ulcerated proximal descending colon (A) Endoscopic image of the transverse colon illustrating deep ulceration with almost complete obliteration of the vasculature; (B) endoscopic image of the transverse colon illustrating superficial ulceration with patchy obliteration of the vasculature; (C) endoscopic image of the ascending colon illustrating erosions, mucosal bleeding, and patchy obliteration of the vasculature

Two years later, the patient developed rectal bleeding and underwent flexible sigmoidoscopy, which showed mild-to-moderate inflammation throughout the rectal stump and was diagnosed as likely diversion colitis. She was managed conservatively as an outpatient with short-chain fatty acid enemas for four weeks. A few months later, she presented to the gastroenterology department with abdominal pain, vomiting, and high stoma output. The differential diagnoses included infectious colitis and inflammatory bowel disease (IBD). CT of the abdomen showed colitis of the distal colon with stomal cellulitis (Figures 2, 3). Flexible sigmoidoscopy of the upstream colon demonstrated moderate-to-severe inflammation and ulceration up to the splenic flexure (Figure 4). Biopsies confirmed acute or chronic marked active inflammation and ulceration with normal crypt architecture. Fecal testing for *Clostridium difficile*, microbiology, and carbapenem-resistant organisms yielded negative results.

The patient received oral ciprofloxacin and intravenous hydrocortisone for five days. Importantly, her symptoms rebounded with worsening per-stoma pus and mucus discharge, along with a raised serum C-reactive protein level of 23 mg/L when intravenous hydrocortisone was switched to oral prednisolone. Given her history of PE and a family history of venous thromboembolism (VTE), vedolizumab, a gut-specific agent with a low VTE risk, was considered. Following further steroid therapy, the patient's stoma output significantly improved, with no further bleeding, pus, or mucus. There was also a significant improvement in the skin irritation around the hernia site. She was then discharged with appropriate follow-up in the IBD clinic.

## Discussion

In summary, our case describes an 82-year-old woman who presented with diversion colitis two years following a Hartmann's procedure and end colostomy and was subsequently diagnosed with UC. While the exact cause and pathophysiology of UC remain unknown, it is likely multifactorial, involving genetic predispositions, autoimmune pathology, epithelial barrier defects, and environmental factors.

Lim et al. [2] described three cases of patients who developed UC in the proximal in-stream colon following diversion colitis after end colostomy. The first patient required post-anal repair due to complications from type-1 diabetes; the second patient had a neoanal canal created due to an imperforate anus; and the third patient underwent small bowel resection for volvulus. These cases indicate that diversion colitis could be a risk factor for UC and that inflammation in a defunctioned colonic segment may trigger pre-stomal UC. They considered whether the removal of the diverted colon could be an effective treatment for UC, but resection was not deemed appropriate in these cases, leaving the question open. Jowett and Cobden [3] and Yaguchi et al. [4] each detailed another case of UC developing following diversion colitis after end colostomy, further supporting this association. Jowett and Cobden's patient required anterolateral repair of the anal sphincter secondary to fecal and urinary incontinence, while Yaguchi's patient had Hartmann's surgery due to a perforated sigmoid diverticulum, which was similar to our case. Yaguchi and colleagues [4] considered whether local inflammation could act as a trigger for UC through lymphocytes or autoantibodies circulating via a lymphogenous or hematogenous route.

The management of various cases tended to involve medical treatment of the diversion colitis as the first line. Often, this was sufficient to resolve symptoms [2-4], and surgery was not indicated, which was similar to our patient's treatment. Aminosalicylates (oral, suppositories, and enemas given rectally to act directly on the diverted colon) were used in almost all the cases except in the one we described and Lim's third case [2]. Surgery seemed to be the second-line option for most of the earlier cases where medical management was ineffective. In our case, symptoms from both the diverted colon and the subsequent upstream UC were refractory to steroids; hence, advanced therapy was considered. This may differ from other case reports, given the wide array of effective advanced therapies now available. Case details, including patient demographics, clinical course, medical treatment, and outcomes, are summarized below (Table 1).

**Table 1:** Previous case reports with patient demographics, clinical course, treatment, and outcome

Case No.	Author (Year)	Patient Demographics	Clinical Course	Medical Treatment for UC	Outcome
1	Lim AG et al. (1999) [2]	60-year-old Caucasian woman; non-smoker	Type-1 diabetes with complications, post-anal repair leading to sigmoid end colostomy, followed by in-stream colonic UC	Oral prednisolone 30 mg (tapered over four months), oral mesalazine 800 mg three times daily, mesalazine foam enema 1g once daily	Improved and remained well
2	Lim AG et al. (1999) [2]	24-year-old Caucasian male; non-smoker	Born with imperforate anus, treated with colostomy. At 16, underwent multiple surgeries to create a neoanal canal with an electrical stimulator and had a defunctioning loop ileostomy. Experienced rectal bleeding and mucus passage, treated as diversion colitis without improvement	Oral prednisolone	Despite treatment with prednisolone, his symptoms worsened, necessitating several additional surgeries, including a sigmoid loop colostomy and later a colectomy, which ultimately led to a good recovery and cessation of steroid therapy
3	Lim AG et al. (1999) [2]	45-year-old woman; non-smoker; strongly cANCA positive	Left-end colostomy for severe diarrhea post-small-bowel resection for volvulus. New recurrent diarrhea, initially treated as diversion colitis, later diagnosed as in-stream colonic UC	No medical treatment	Underwent ileostomy with excision of the in-stream colon and rectal stump. Outcome unknown
4	Jowett SL et al. (2000) [3]	75-year-old female; ex-heavy smoker; mother had unspecified colitis	Traumatic forceps delivery at age 30. Experienced fecal and urinary incontinence, underwent anterolateral repair of the anal sphincter and end colostomy without symptom improvement. New blood and mucus per rectum were treated as diversion colitis. Symptoms worsened; new in-stream colonic UC was diagnosed	Oral mesalazine and topical steroids per stoma	Improvement in symptoms
5	Yaguchi K et al. (2022) [4]	63-year-old male	Hartmann’s operation following perforation of a sigmoid colon diverticulum and peritonitis. Stoma closure left a portion of the sigmoid colon with diverticulum unintentionally. New blood per rectum, treated as diversion colitis with intermittent use of topical mesalazine enemas. Later developed UC in the rectum and blind sigmoid colon	Mesalazine suppositories and mesalazine enemas	No relapse since remission

The British Society of Gastroenterology guidelines [5] state that the initial therapy for adults admitted with acute severe UC should be intravenous (IV) hydrocortisone to induce remission. Should this fail, a surgical review for colectomy is warranted. If a colectomy is not required, then second-line therapy would be IV infliximab or IV ciclosporin. In our case, the patient responded well to a five-day course of IV hydrocortisone, demonstrating initial symptom improvement. This led to a tapering course of oral prednisolone. However, her symptoms recurred with a rapid weaning schedule, necessitating a prolonged tapering course. Given her comorbidities and the desire to avoid azathioprine combination therapy with infliximab, she was offered long-term maintenance therapy with vedolizumab.

Key learning points include recognizing that patients can develop UC on a background of diversion colitis following bowel surgeries. There may be a mechanistic explanation for this, and standard UC management approaches should be used unless specific patient factors indicate that these patients are more treatment-refractory than those with typical UC. Currently, there is a lack of evidence as to whether this patient group should be treated differently from the standard UC cohort. Further research is required to monitor complications post-end colostomies. Retrospective or prospective data collection on the prevalence of developing diversion colitis or UC post-end colostomies is needed, with larger sample sizes than previous reports, including ours.

## Conclusions

This case adds further support to the hypothesis that diversion colitis may predispose patients to the development of de novo UC. We recommend considering UC as a differential diagnosis, even in older patients who present with typical symptoms such as diarrhea, rectal bleeding, and abdominal pain, as well as atypical signs such as changes in stoma integrity and delayed healing in those with a colostomy who have developed diversion colitis. Additionally, we advocate for shared decision-making with patients, particularly among the elderly, as this approach may enhance treatment compliance.
